# Metabolic engineering for the high-yield production of isoprenoid-based C_5_ alcohols in *E. coli*

**DOI:** 10.1038/srep11128

**Published:** 2015-06-08

**Authors:** Kevin W. George, Mitchell G. Thompson, Aram Kang, Edward Baidoo, George Wang, Leanne Jade G. Chan, Paul D. Adams, Christopher J. Petzold, Jay D. Keasling, Taek Soon Lee

**Affiliations:** 1 Joint BioEnergy Institute, Emeryville, CA 94608, USA; 2 Department of Plant and Microbial Biology, University of California, Berkeley, CA 94720, USA; 3 Department of Plant and Microbial Biology, University of California, Berkeley, CA 94720, USA; 4 Department of Bioengineering, University of California, Berkeley, CA 94720, USA; 5 Department of Chemical & Biomolecular Engineering, University of California, Berkeley, CA 94720, USA

## Abstract

Branched five carbon (C_5_) alcohols are attractive targets for microbial production due to their desirable fuel properties and importance as platform chemicals. In this study, we engineered a heterologous isoprenoid pathway in *E. coli* for the high-yield production of 3-methyl-3-buten-1-ol, 3-methyl-2-buten-1-ol, and 3-methyl-1-butanol, three C_5_ alcohols that serve as potential biofuels. We first constructed a pathway for 3-methyl-3-buten-1-ol, where metabolite profiling identified NudB, a promiscuous phosphatase, as a likely pathway bottleneck. We achieved a 60% increase in the yield of 3-methyl-3-buten-1-ol by engineering the Shine-Dalgarno sequence of *nudB*, which increased protein levels by 9-fold and reduced isopentenyl diphosphate (IPP) accumulation by 4-fold. To further optimize the pathway, we adjusted mevalonate kinase (MK) expression and investigated MK enzymes from alternative microbes such as *Methanosarcina mazei*. Next, we expressed a fusion protein of IPP isomerase and the phosphatase (Idi1~NudB) along with a reductase (NemA) to diversify production to 3-methyl-2-buten-1-ol and 3-methyl-1-butanol. Finally, we used an oleyl alcohol overlay to improve alcohol recovery, achieving final titers of 2.23 g/L of 3-methyl-3-buten-1-ol (~70% of pathway-dependent theoretical yield), 150 mg/L of 3-methyl-2-buten-1-ol, and 300 mg/L of 3-methyl-1-butanol.

There has been considerable interest in the biosynthetic production of C_2_ – C_5_ alcohols as commodity chemicals and potential biofuels^1^,^2^. Butanol, for instance, serves as a platform chemical for thousands of compounds and can be used as a biogasoline or fuel additive^3^,^4^. Some of the most promising work in the microbial synthesis of these compounds has used refactored amino acid biosynthesis pathways to produce a variety of C_3_ – C_5_ alcohols at high titers^5^. Using this method, valuable alcohols such as isobutanol (C_4_) have been produced at impressively high yields and titers^6^.

Isoprenoid biosynthesis provides an additional route to energy-dense C_5_ alcohols, namely isopentenol (3-methyl-3- and 3-methyl-2-buten-1-ol, also known as isoprenol and prenol, respectively) and isopentanol (3-methyl-1-butanol)^7^. These alcohols have octane numbers and combustion properties that make them potential gasoline replacements^8^. Isopentanol, for example, has been evaluated for use in homogenous charge compression ignition (HCCI) engines and significantly outperforms ethanol^9^. In addition, these alcohols were shown to function as ideal anti-knock additives in spark ignition engines^10^. Although isoprenoids can be produced from both the methylerythritol phosphate (MEP) and mevalonate (MVA) pathways, initial engineering efforts towards isopentenol production have focused primarily on the MVA pathway^11^,^12^,^13^,^14^. Thus far, alcohol yields from these efforts compare favorably with analogous work using the MEP pathway^15^.

C_5_ alcohols can be produced from the MVA pathway following the dephosphorylation of isopentenyl diphosphate (IPP) and dimethylallyl diphosphate (DMAPP), the universal precursors of all isoprenoid compounds. A specific protein capable of catalyzing this dephosphorylation, NudF from *B. subtilis*, was first identified using a screening method based on prenyl diphosphate toxicity^11^. In a later study, an *E. coli* native enzyme (NudB) was also shown to effectively catalyze the dephosphorylation of IPP and DMAPP^12^. When paired with the IPP-overproducing mevalonate pathway, the expression of NudB produced 3-methyl-3-buten-1-ol at 8.3% theoretical yield. Using a fusion protein of IPP isomerase (Idi1) and NudB, IPP-derived 3-methyl-3-buten-1-ol and DMAPP-derived 3-methyl-2-buten-1-ol were produced concurrently. With the expression of an *E. coli-*native reductase (NemA), 3-methyl-2-buten-1-ol was successfully reduced to 3-methyl-1-butanol, albeit at low efficiency^12^.

Although optimization of the heterologous MVA pathway has improved the production of 3-methyl-3- and 3-methyl-2-buten-1-ol, yields are still considerably lower than those required for large-scale, economical production^13^,^14^. Furthermore, optimization towards the production of fully reduced 3-methyl-1-butanol has not been attempted. In the current work, we engineered the heterologous MVA pathway in *E. coli* to achieve the highest yields yet reported for 3-methyl-3-buten-1-ol. In addition, we improved the production of 3-methyl-1-butanol by more than 10-fold over previous results.

## Results and Discussion

### Pathway organization and engineering strategy

**Figure 1:**
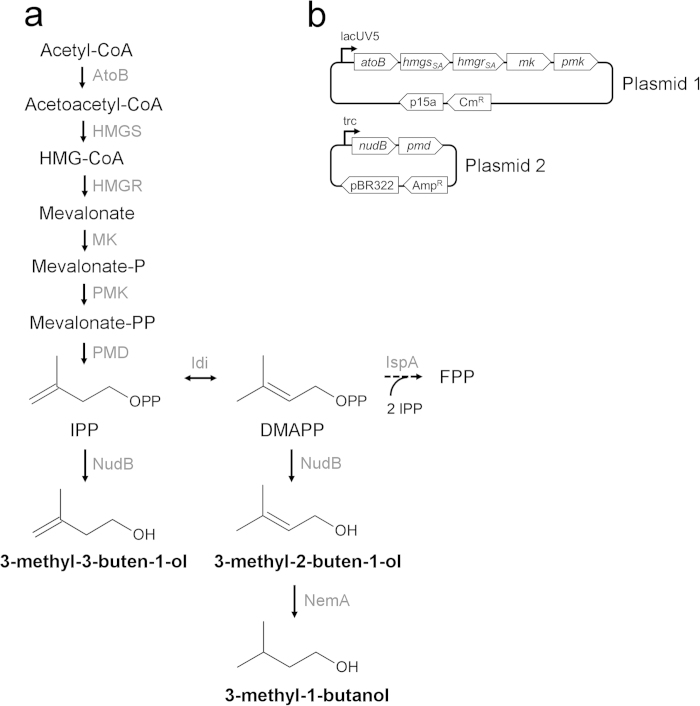
A heterologous MVA pathway for C5 alcohol production. (a) Pathway overview. The heterologous mevalonate pathway in *E. coli* consists of 7 reactions to convert acetyl-CoA into IPP and DMAPP. Dephosphorylation of these compounds by NudB, a promiscuous *E. coli* phosphatase, produces 3-methyl-3-buten-1-ol and 3-methyl-2-buten-1-ol, respectively. NemA, an endogenous reductase, is capable of reducing 3-methyl-2-buten-1-ol, but not 3-methyl-3-buten-1-ol, into fully reduced 3-methyl-1-butanol. **(b)** Plasmid architecture. A two plasmid system for 3-methyl-3-buten-1-ol production served as the initial engineering platform (strain KG1). Plasmid 1 contained genes from *atoB* to *PMK* with a medium copy p15A ori and weak lacUV5 promoter^16^. Plasmid 2 contained *nudB* and *PMD* with a high copy pBR322 ori and strong trc promoter. To produce mixed alcohols, a fusion protein and reductase were expressed on plasmid two (see Fig. 4).

The heterologous MVA pathway in *E. coli* (Fig. 1a) allows for the biosynthesis of IPP and DMAPP, the universal precursors to all isoprenoid compounds. To produce 3-methyl-3-buten-1-ol from IPP, *nudB,* a gene encoding an *E. coli* native phosphatase, is overexpressed in addition to the mevalonate pathway. If an isomerase such as *IDI1* from yeast is expressed, DMAPP and its corresponding alcohol 3-methyl-2-buten-1-ol will be formed. Expression of a reductase such as *nemA* will convert 3-methyl-2-buten-1-ol, but not 3-methyl-3-buten-1-ol, into fully reduced 3-methyl-1-butanol^12^ (Fig. 1a).

We initially assembled a two-plasmid system for 3-methyl-3-buten-1-ol production: plasmid 1 (pJBEI-6829), a medium copy vector with a lacUV5 promoter (pBbA5c^16^), contained mevalonate pathway genes from thiolase (*atoB*) to phosphomevalonate kinase (*PMK*) while plasmid 2 (pJBEI-6833), a high copy vector with a trc promoter (pTrc99A^17^), contained *nudB* and diphosphomevalonate decarboxylase (*PMD*) (Fig. 1b) (Table 1). In this pathway, *atoB* and *nudB* were derived from *E. coli*, *mvaS* and *mvaA*, which encode 3-hydroxy-3-methylglutaryl-CoA (HMG-CoA) synthase and reductase, respectively, were from *Staphylococcus aureus*^18^, and *MK*, *PMK*, and *PMD* were derived from yeast. These plasmids were transformed into *E. coli* DH1 to create strain KG1, which was used as an initial platform for subsequent engineering^14^.

Our engineering strategy was to first optimize 3-methyl-3-buten-1-ol production before moving towards the production of mixed C_5_ alcohols. Rather than focusing on high-throughput strain generation or combinatorial pathway assembly, we applied robust metabolomics and proteomics methods to identify likely bottlenecks and rationally direct pathway engineering. Once we optimized the precursor pathway and improved 3-methyl-3-buten-1-ol production, we implemented additional engineering to produce 3-methyl-2-buten-1-ol and ultimately fully reduced 3-methyl-1-butanol.

### Metabolite profiling of KG1 identifies IPP accumulation as a likely bottleneck

Strain KG1 produced ~1.2 g/L of 3-methyl-3-buten-1-ol on 1% glucose after 48 hours, equivalent to 36% of pathway-dependent theoretical yield^14^. While an improvement over the original strain^19^, this titer was well below levels necessary for economical, large-scale production. We hypothesized that one or more pathway bottlenecks were limiting product yields in strain KG1. To accurately identify these bottlenecks, we performed a comprehensive analysis of pathway metabolites and proteins during the fermentation time-course.

**Figure 2:**
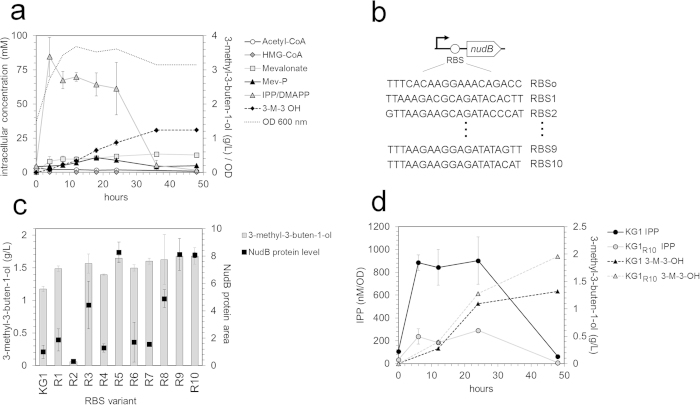
RBS engineering to improve 3-methyl-3-buten-1-ol titer and reduce IPP accumulation. **(a)** Metabolite profiling of strain KG1. Mevalonate pathway intermediates were quantified in strain KG1 over a 48 hour time-course by LC-MS, showing IPP as the highest accumulating intermediate by a factor of 8. A growth curve for strain KG1 is shown as a dashed line. Error bars represent standard deviation (n = 3). 3-M-3-OH = 3-methyl-3-buten-1-ol. **(b)** RBS optimization of ***nudB***. Ten RBS variants of *nudB* (see Table S1) were generated using RBS calculator and cloned into plasmid 2, forming JPUB-004498 to JPUB-004507. **(c)** 3-Methyl-3-buten-1-ol titer and NudB protein level in KG1_R1_ – KG1_R10_. Strains containing nudB_RBS1_ through nudB_RBS10_ (KG1_R1_ - KG1_R10_) were assayed for 3-methyl-3-buten-1-ol and NudB protein level. Bars represent 3-methyl-3-buten-1-ol titer after 36 hours. Squares show NudB protein area at 24 hours relative to that measured in strain KG1 (normalized to a value of 1). Error bars represent standard deviation (n = 3). **(d)** IPP accumulation and 3-methyl-3-buten-1-ol production in strains KG1 and KG1_**R10**_. IPP concentrations in KG1_R10_ were reduced by 4-fold relative to KG1, while 3-methyl-3-buten-1-ol titer increased by 60%. Error bars represent standard deviation (n = 3).

Quantification of MVA pathway intermediates in strain KG1 over a 48 hour time-course (Fig. 2a) revealed that IPP concentrations exceeded those of any other observed MVA pathway intermediate by more than 8-fold. The comparatively low concentrations of acetyl-CoA, HMG-CoA, mevalonate, and mevalonate phosphate suggested that flux through the upstream pathway was unlikely to be limiting. The accumulation of IPP alone implied that NudB, the protein responsible for the conversion of IPP into 3-methyl-3-buten-1-ol, was the primary bottleneck in strain KG1. IPP has previously been shown to be toxic, resulting in growth inhibition and a reduction in glucose uptake at high concentrations. Although no obvious growth defects were apparent in strain KG1, more subtle effects such as feedback inhibition^20^ could be deleterious to host and the pathway function. Even without exerting toxicity, the accumulation of a metabolite upstream of 3-methyl-3-buten-1-ol indicated suboptimal pathway performance. Consequently, reducing IPP accumulation was a priority in further engineering.

Quantification of pathway proteins in strain KG1 (Supplemental Figure S1) was conducted with targeted proteomics based on selected reaction monitoring (SRM)^21^,^22^ to assess protein stability and relative concentrations over a 48-hour time-course. In general, pathway proteins appeared stable throughout the fermentation other than the expected increase following pathway induction (0 to 6 h). Although this SRM method reports relative peak areas for each protein rather than absolute concentrations, low signal intensity often indicates poor protein expression and potential pathway bottlenecks^14^,^22^,^23^. Peak area was the lowest for HMG-CoA reductase (HMGR), implying weak expression of this enzyme, but the low steady-state levels of HMG-CoA (the substrate of HMGR) and rapid accumulation of IPP (a downstream product after the HMGR-catalyzed reaction) suggested that HMGR may not be limiting under current conditions. Protein areas of PMD and NudB, both of which were expressed from a high-copy plasmid, were an order of magnitude above the other pathway proteins. Although NudB is an *E. coli* native protein, the plasmid-borne copy was the primary source of quantifiable protein; background levels of NudB in DH1 wild type were more than 10-fold less than strain KG1 (data not shown).

### Improved NudB protein expression reduces IPP accumulation and yields high 3-methyl-3-buten-1-ol titers

To prevent the accumulation of IPP, we could either slow down its formation through upstream pathway engineering or enhance its conversion to 3-methyl-3-buten-1-ol by improving NudB-catalyzed reaction efficiency. Since we did not want to decrease upstream pathway efficiency, we focused on improving flux to 3-methyl-3-buten-1-ol by increasing NudB expression. To increase NudB protein levels, we optimized the Shine-Dalgarno sequence of *nudB* using the RBS calculator^24^. Starting with the RBS sequence in strain KG1 (NudB_RBSo_) as an input, 10 sequences were generated that were predicted to improve NudB protein expression (Fig. 2b). We designed the 10 RBS sequences (RBS1 – RBS10) to achieve a range of expression to allow for the titration of NudB levels in subsequent engineering (Supplemental Table 1). After cloning these variants into plasmid 2, we co-transformed each of them into *E. coli* DH1 with pJBEI-6829 to generate strains KG1_R1_ (strain KG1 containing NudB_RBS1_) through KG1_R10_ (strain KG1 containing NudB_RBS10_) (Table 1). We performed a production test with these strains and collected protein samples to quantify levels of NudB (Fig. 2c).

Protein levels of NudB were improved in all RBS sequence variants with the notable exception of NudB_RBS2_. The best-performing RBS sequences, RBS5, RBS9, and RBS10, yielded NudB protein levels 9-fold higher than the original sequence (Fig. 2c). As anticipated, strains with improved NudB expression also produced more 3-methyl-3-buten-1-ol (Fig. 2c). Surprisingly, the variability in 3-methyl-3-buten-1-ol titer was minor compared to the large changes in NudB protein level; even the slight increase in NudB protein in strain KG1_R7_ yielded a 3-methyl-3-buten-1-ol titer comparable to strain KG1_R10_, which produced 8-fold more NudB. This observation suggested that NudB expression was no longer limiting in the highest-performing strains, which produced ~1.6 g/L of isopentenol at 36 hours. Indeed, expression of additional NudB on a supplemental plasmid yielded no additional improvement in titer (Supplemental Figure S2). Still, we chose KG1_R10_ for further study, reasoning that this strain provided room for additional improvement in the precursor pathway and increased flux to IPP.

Metabolite analysis of KG1_R10_ confirmed that improved NudB expression relieved IPP accumulation. Over a 48-hour time-course, IPP levels in strain KG1_R10_ were reduced by 4-fold relative to KG1 (Fig. 2d). At 48 hours, the 3-methyl-3-buten-1-ol titer in strain KG1_R10_ reached 1.94 g/L, a 60% increase over strain KG1. Given the significant reduction in IPP level observed in strain KG1_R10_, we suspected that the pathway bottleneck might have shifted from NudB reaction to the upstream precursor pathway. If this were the case, further increases in IPP production could yield additional increases in 3-methyl-3-buten-1-ol titer.

### Optimization of MK yields minor improvements in 3-methyl-3-buten-1-ol titer

Although the 3-methyl-3-buten-1-ol pathway consists of seven genes, MK is the primary determinant of flux to IPP^14^. Sufficient flux to IPP is required for high product yields, but excessive MK expression can lead to IPP accumulation and a reduction in growth and 3-methyl-3-buten-1-ol titer. Since we increased the “pull” on IPP using NudB_RBS10_ (Fig. 2d), we decided to increase the “push” to IPP through additional MK engineering. We employed two approaches: 1) pairing improved NudB expression with increased expression of MK, and 2) introducing MK homologs with desirable properties from other organisms. In each case, the *MK* gene was positioned downstream of *PMK* as this gene order previously increased MK protein levels^14^.

**Figure 3:**
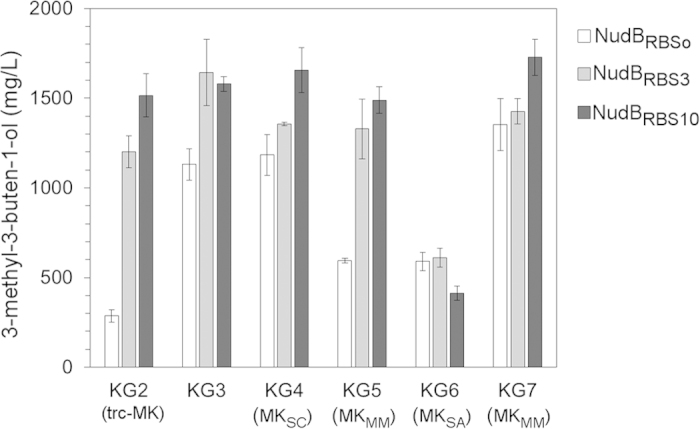
Altering MK expression and identity to increase flux to IPP. To increase pathway flux to IPP, MK expression and identity was altered and evaluated with different concentrations of NudB (see table 1 for a list of strains). 3-Methyl-3-buten-1-ol titers are shown at 36 hours post-induction. Error bars show standard deviation (n = 3).

MK expression was enhanced by inserting a trc promoter upstream of *MK* (pJBEI-6832), which was shown to increase MK protein levels by ~4-fold^14^. We paired this plasmid with the previously characterized plasmids containing NudB_RBSo_ (creating strain KG2), NudB_RBS3_ (strain KG2_R3_), and NudB_RBS10_ (strain KG2_R10_) to titrate low, medium, and high levels of NudB protein, respectively (Fig. 3). We compared strain KG2 with the control strain KG3, which contained an identical version of the pathway with the same gene order but no supplemental promoter (Table 1). 3-Methyl-3-buten-1-ol titer in KG2 was 268 mg/L, a 4-fold reduction compared to KG3. If this low titer was due to IPP accumulation, we anticipated that elevated NudB levels would dramatically increase 3-methyl-3-buten-1-ol titer. In accordance with our expectation, strain KG2_R3_, which expressed a higher level of NudB, produced 4-fold more 3-methyl-3-buten-1-ol than strain KG2. Even with this improvement, however, titers were about 20% less than the comparable control (strain KG3_R3_). With NudB_RBS10_ (i.e. strain KG2_R10_), we recorded an additional increase in titer to 1550 mg/L of 3-methyl-3-buten-1-ol, matching the performance of the control KG3_R10_ (Fig. 3). Attempts to further increase NudB levels with an additional plasmid (pBbB8k-NudB, pJBEI-6835) were unsuccessful (Supplemental Figure S2), suggesting that IPP accumulation was no longer problematic in strain KG2_R10_. Although elevated MK expression appeared to increase flux to IPP, the strong performance of the control strain suggested it was not necessary for peak pathway efficiency. Consequently, we focused on changing MK identity rather than expression level for further optimization.

Although most work on MVA pathway optimization has focused on *S. cerevisiae*-derived MK (MK_SC_), MK enzymes from alternative hosts such as *Staphylococcus aureus* (MK_SA_) and the archaea *Methanosarcina mazei* (MK_MM_) have promising characteristics. MK_MM_, for example, was shown to resist feedback inhibition from IPP, GPP, and FPP^20^. We tested these MK variations with a MVA pathway “top” portion that provided high flux to mevalonate (MevTco)^14^, reasoning that this context could better reveal differences in MK activity. Strains KG4, KG5, and KG6 contained this high flux pathway paired with MK enzymes derived from *S. cerevisiae*, *M. mazei*, and *S. aureus*, respectively (Table 1). As practiced above, we titrated each pathway with low (NudB_RBSo_), medium (NudB_RBS3_), and high (NudB_RBS10_) levels of NudB.

With low levels of NudB (i.e. NudB_RBSo_), MK_SC_ outperformed MK_MM_ and MK_SA_: strain KG4 produced 2-fold more 3-methyl-3-buten-1-ol at 36 hours than KG5 and KG6 (Fig. 3). Surprisingly, growth inhibition consistent with IPP accumulation was clearly observed in strain KG5 (Supplemental Figure S3). This observation implied that the flux to IPP was actually increased when using MK_MM_. Consistent with this expectation, increased NudB expression by RBS engineering relieved the growth inhibition in strain KG5 (Supplemental Figure S3) and doubled 3-methyl-3-buten-1-ol production (Fig. 3). Despite this increase, maximum titers matched those of strain KG4, which produced 1650 mg/L of 3-methyl-3-buten-1-ol when paired with NudB_RBS10_. Surprisingly, 3-methyl-3-buten-1-ol production in strain KG6 (containing MK_SA_) did not increase with additional NudB protein. This observation suggested that *S. aureus* MK did not provide adequate pathway flux to IPP.

Since the preliminary results for MK_MM_ were promising, we investigated this homolog further by pairing the enzyme with a different MVA pathway “top” portion, MevT_SA_, to create strain KG7 (Table 1). In this pathway context, a maximum 3-methyl-3-buten-1-ol titer of 1730 mg/L was recorded with NudB_RBS10_ (Fig. 3). Although MK_MM_ did appear to improve flux to IPP relative to MK_SC_, similar maximum titers were reached with each enzyme. This apparent “plateau” in 3-methyl-3-buten-1-ol titer may suggest that further gains from MVA pathway engineering will be difficult to achieve. Although NudB protein expression is no longer a bottleneck, the poor kinetics of the enzyme^12^ may play a key role in limiting 3-methyl-3-buten-1-ol production. It is possible that an improvement in NudB kinetics is required to facilitate additional titer increases in the current pathway context.

### Expression of Idi1~NudB fusion protein and NemA results in the production of 3-methyl-3-buten-1-ol, 3-methyl-2-buten-1-ol, and 3-methyl-1-butanol

**Figure 4:**
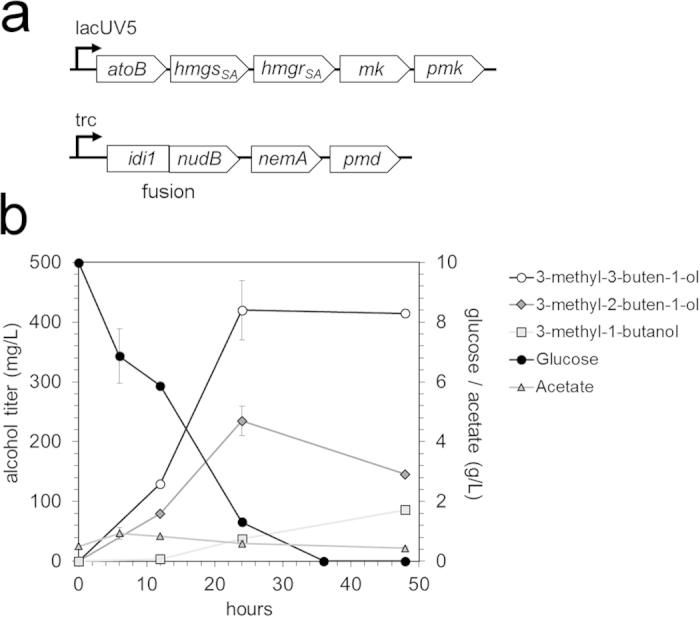
Engineering to produce mixed alcohols. **(a) Plasmid architecture.** To produce 3-methyl-2-buten-1-ol and 3-methyl-1-butanol in addition to 3-methyl-3-buten-1-ol, plasmid 1 from strain KG1 (Cm^R^, p15A ori) was paired with a mixed alcohol production plasmid (Amp^R^, pBR322 ori) encoding an Idi1~NudB fusion protein and a reductase (NemA)^12^ to create strain KG8. **(b)** Mixed alcohol production in strain KG8. A fermentation time-course revealed that strain KG8 produced three C_5_ alcohols. By 48 hours, strain KG8 produced over 400 mg/L of 3-methyl-1-buten-1-ol, 150 mg/L of 3-methyl-2-buten-1-ol, and 100 mg/L of 3-methyl-1-butanol. All glucose was consumed by 36 hours, and acetate secretion was minimal. Error bars represent standard deviation (n = 3).

Since we successfully optimized a strain for 3-methyl-3-buten-1-ol production, we next sought to improve the production of 3-methyl-2-buten-1-ol and subsequently 3-methyl-1-butanol. To produce these alcohols, we over-expressed the yeast-derived IPP isomerase Idi1 and the *E. coli-*derived promiscuous reductase NemA to create strain KG8 (Table 1). Idi1 and NudB were expressed together as a fusion protein as described previously^12^ (Fig. 4a).

Strain KG8 produced 3-methyl-3-buten-1-ol, 3-methyl-2-buten-1-ol, and fully reduced 3-methyl-1-butanol as anticipated (Fig. 4b). At 48 hours, total C_5_ alcohol content (i.e. the summed total 3-methyl-3-buten-1-ol, 3-methyl-2-buten-1-ol, and 3-methyl-1-butanol) reached 700 mg/L, a 4-fold improvement in titer over the previously reported result^12^. From 24 to 48 hours, the increase in 3-methyl-1-butanol production appeared to correlate with the reduction of 3-methyl-2-buten-1-ol as expected given its route of formation (Fig. 1a).

**Figure 5:**
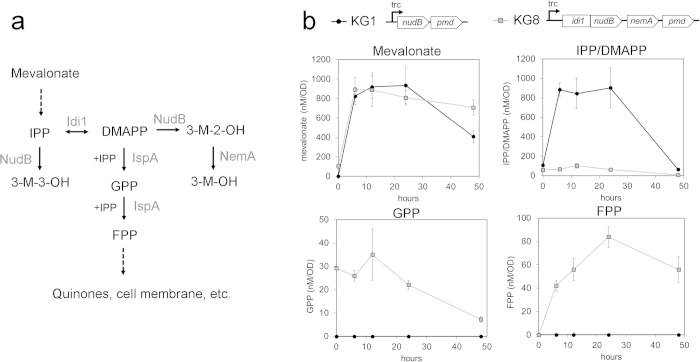
Metabolite profiling of strain KG8 reveals competition with endogenous pathways. **(a) Competition with endogenous pathways.** Expression of yeast Idi1 increases flux to DMAPP and subsequently the longer chain terpenes GPP and FPP. Competition between 3-methyl-2-buten-1-ol and these terpenes likely limits alcohol production. 3-M-3-OH = 3-methyl-3-buten-1-ol. 3-M-2-OH = 3-methyl-2-buten-1-ol. 3-M-OH = 3-methyl-1-butanol. **(b)**Detection of GPP and FPP in strain KG8. GPP and FPP were detected in mixed alcohol strain KG8, but not in strain KG1, the 3-methyl-3-buten-1-ol production strain. Although downstream metabolite concentrations varied substantially, steady state levels of mevalonate were nearly identical, suggesting that flux through the upstream pathway was not affected. Error bars show standard deviation (n = 3).

Compared to strain KG1, total C_5_ alcohol content in KG8 decreased by over 40%. We hypothesized that competition with endogenous pathways was responsible for this reduction in overall titer. We suspected that expression of Idi1—required for DMAPP and 3-methyl-2-buten-1-ol production—would divert carbon towards the formation of geranyl diphosphate (GPP) and farnesyl diphosphate (FPP), both of which are used in a variety of cellular processes (Fig. 5a). Consistent with this hypothesis, GPP and FPP were observed in strain KG8, but below detection in the 3-methyl-3-buten-1-ol production strain KG1 (Fig. 5b). A 10-fold decrease in the concentration of IPP/DMAPP was also observed in KG8, likely reflecting the multiple routes of IPP/DMAPP depletion in this strain. The detection of GPP and FPP in strain KG8 makes *E. coli* FPP synthase (*ispA*) an attractive engineering target; it is likely that reducing or eliminating IspA activity will yield increased production of 3-methyl-2-buten-1-ol and subsequently 3-methyl-1-butanol. Though levels of IPP, GPP, and FPP varied significantly between KG8 and KG1, mevalonate concentrations were similar in both strains. This suggested that carbon flow through the upstream mevalonate pathway was unaffected by the implementation of downstream engineering.

**Figure 6:**
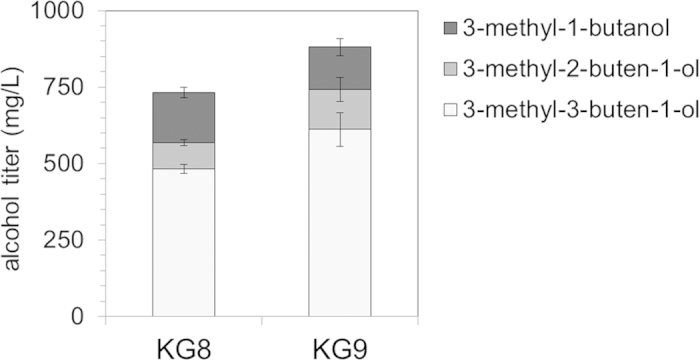
Increased NudB expression improves total C5 alcohol content. The introduction of a 3^rd^ plasmid containing an additional copy of *nudB* (pBbB8k-NudB, see Table 1) in strain KG9 yielded a 20% increase in total alcohol content at 48 hours post induction. Error bars show standard deviation (n = 3).

We increased the level of NudB protein to optimize the pathway given its positive effect in strain KG1 (Fig. 2). Since NudB in strain KG8 was part of a protein fusion with Idi1, we increased NudB levels by using a third plasmid, pBbB8k-NudB (pJBEI-6835)^14^. We designated this 3-plasmid strain as KG9 (Table 1). Compared to strain KG8, total mixed alcohol content at 48 hours increased more than 20% to 880 mg/L (Fig. 6). However, 3-methyl-3-buten-1-ol accounted for the entirety of this increase—levels of 3-methyl-2-buten-1-ol remained constant while those of 3-methyl-1-butanol slightly decreased. More engineering is clearly required to direct carbon flow from DMAPP towards 3-methyl-2-buten-1-ol and 3-methyl-1-butanol and away from GPP and FPP. One promising approach would be the development or discovery of a phosphatase specific for DMAPP. Although NudB is effective in producing high titers of C_5_ alcohol, the enzyme is promiscuous and does not appear to discriminate between IPP or DMAPP, both of which are non-native substrates^25^. More broadly, carbon loss to GPP and FPP should be mitigated through a selective knockdown of IspA activity. This may prove challenging, however, since the formation of FPP and thus *ispA* is essential for *E. coli* growth^26^.

### An oleyl alcohol overlay improves yields of each C_5_ alcohol

Since short-chain alcohols and terpenes are often volatile, calculated yields are commonly underestimated during fermentation. In the production of 3-methyl-3-buten-1-ol, an apparent decrease in recoverable alcohol observed from 48 to 72 hours (Supplemental Figure S4) suggested that evaporation was occurring. To quantify the amount of 3-methyl-3-buten-1-ol, 3-methyl-2-buten-1-ol, and 3-methyl-1-butanol lost during production assays, we spiked each alcohol into culture tubes containing growth medium at various concentrations and monitored recovery over time at 30°C (Supplemental Figure S5). After 48 hours, losses of 20%, 10%, and 40% were observed for 3-methyl-3-buten-1-ol, 3-methyl-2-buten-1-ol, and 3-methyl-1-butanol, respectively. When the same tubes were incubated at 4°C, 100% recovery of each alcohol was observed after 48 hours (data not shown).

**Figure 7:**
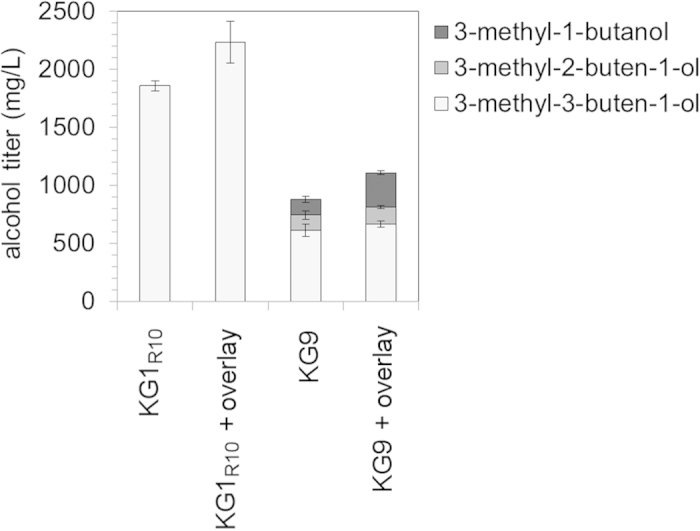
An oleyl alcohol overlay improves C5 alcohol recovery. The addition of a 20% overlay of oleyl alcohol increased the titers of strains KG1_R10_ and KG9 by 20% and 26%, respectively. Concentrations at 48 hours post induction are shown. Error bars represent standard deviation (n = 3).

Hydrophobic overlays such as decane or dodecane are often used to prevent evaporation during microbial fermentations, particularly in the case of fatty acids or longer chain isoprenoids^27^,^28^. For the recovery of alcohols such as 1-butanol or 3-methyl-1-butanol, however, oleyl alcohol has proven a better choice in both *Clostridia*^29^ and *E. coli*^30^. We assessed the efficacy of an oleyl alcohol overlay with KG1_R10_ and KG9, high producers of 3-methyl-3-buten-1-ol and mixed alcohols, respectively. Using a 20% oleyl alcohol overlay (10 mL overlay added to 50 mL of culture) with strain KG1_R10_, a 3-methyl-3-buten-1-ol titer of 2.23 g/L (70% theoretical) was recorded after 48 hours, a 20% increase over the same strain without an overlay (Fig. 7). With strain KG9, an overlay led to a 26% increase in total C_5_ alcohol content (Fig. 7). The largest increase was in 3-methyl-1-butanol, where titers more than doubled to ~300 mg/L. The increased volatility of 3-methyl-1-butanol relative to 3-methyl-3- and 3-methyl-2-buten-1-ol (Supplemental Figure S5) and differential partitioning of 3-methyl-1-butanol into oleyl alcohol (Supplemental Figure S6) provide potential explanations for the improved recovery of this alcohol. We suspected this was due to the increased volatility (Supplemental Figure S5) and partitioning (Supplemental Figure S6) of this alcohol relative to 3-methyl-3- and 3-methyl-2-buten-1-ol.

## Conclusions

In this work we report the successful metabolic engineering of *E. coli* for the production of three isoprenoid-derived C_5_ alcohols: 3-methyl-3-buten-1-ol, 3-methyl-2-buten-1-ol, and 3-methyl-1-butanol. Using targeted metabolomics and proteomics, we rapidly identified pathway bottlenecks and improved titer. This approach was particularly successful for 3-methyl-3-buten-1-ol, where final yields approached 70% of pathway-dependent theoretical maximum. To achieve high C_5_ alcohol yields from high flux mevalonate pathway strains and prevent IPP accumulation, efficient phosphatase activity was critical. We significantly reduced IPP accumulation through RBS engineering of NudB, achieving a 9-fold improvement in protein level, a 4-fold reduction in IPP levels, and a 60% increase in 3-methyl-3-buten-1-ol production. Although increased NudB expression resulted in high yields in our most productive strains, significant improvements in pathway productivity will be required for fermentation scale-up. Future work to develop a more catalytically active phosphatase should be undertaken to achieve this goal.

Production titers of 3-methyl-2-buten-1-ol and 3-methyl-1-butanol were improved by more than 10-fold compared to previous work, where titers on 0.2% glucose were <10 mg/L^12^. Furthermore, we demonstrated that an oleyl alcohol overlay minimizes product loss due to evaporation and is effective for C_5_ alcohol fermentations. Still, additional engineering is required to improve total C_5_ alcohol content and optimize for the production of 3-methyl-2-buten-1-ol and 3-methyl-1-butanol. The detection of GPP and FPP, metabolites of endogenous isoprenoid metabolism, makes *E. coli* FPP synthase (*ispA*) an attractive engineering target. A reduction in IspA expression or activity should reduce carbon loss to FPP and improve flux to 3-methyl-2-buten-1-ol. Although NemA successfully catalyzes the conversion of 3-methyl-2-buten-1-ol to fully reduced 3-methyl-1-butanol, it does so at a low efficiency^12^. Protein engineering to improve the kinetics of this reaction should result in the complete conversion to 3-methyl-1-butanol. As titers of C_5_ alcohols increase, product toxicity is likely to become an issue^31^. Although use of oleyl alcohol may reduce toxicity, host engineering for increased tolerance^32^ may eventually be required to reach production goals.

## Methods

Chemicals, solvents and media components were purchased and used without modification from Sigma-Aldrich (St. Louis, MO), Fisher Scientific (Pittsburgh, PA), or VWR (West Chester, PA) unless otherwise noted. *E. coli* strains DH10B (Invitrogen, Carlsbad, CA) and DH1 (ATCC) were used for plasmid construction and production experiments, respectively. For targeted proteomics experiments, mass spectrometric-grade trypsin was obtained from Sigma-Aldrich and prepared according to manufacturer's instructions.

### Plasmid and strain construction

*E. coli* DH10B was used as the host for all cloning and plasmid manipulations. The BglBrick standard^33^ was used to assemble all plasmids as previously described. With the exception of pTrc99A^17^, plasmids were derived from the BglBrick plasmid library^16^. *E. coli* DH1 was used as the host for all production assays. Plasmids used in this study are available on the JBEI public registry and listed in Table 1 along with a brief description of production strains.

### Production assays

Starter cultures of *E. coli* DH1 harboring production plasmids were grown overnight in LB medium containing appropriate antibiotics at 37 °C and shaken at 200 rpm in rotary shakers. Chloramphenicol, ampicillin, and kanamycin were provided at final concentrations of 25 mg/L, 100 mg/L, and 25 mg/L, respectively. Production assays were performed in triplicate in EZ-Rich defined medium (Teknova) containing 1% glucose. Briefly, starter cultures were used to inoculate 5 mL of production media in a culture tube or 50 mL of production media in a 250 mL Erlenmeyer flask to an OD_600_ of 0.1. Production cultures were grown in rotary shakers (200 rpm) at 37 °C to an OD_600_ of 0.4-0.6 and induced with 500 μM isopropyl β-D-1-thiogalactopyranoside (IPTG). Strains harboring pJBEI-6835 were also induced with 20 mM arabinose. Following induction, cultures were moved to 30°C for the duration of the assay. At set times, samples were taken for C_5_ alcohol quantification analysis by GC-FID as described previously^12^. For samples containing oleyl alcohol, the entire culture volume was extracted with ethyl acetate after 48 hours. Samples were analyzed by GC-FID as previously described^30^.

### Metabolite quantification

Glucose and organic acids were quantified in filter-sterilized supernatant by high performance liquid chromatography (HPLC) at set time points using an Agilent 1200 Series HPLC system. Intracellular concentrations of mevalonate and IPP were measured by liquid chromatography mass spectrometry (LC-MS). Please see references 14 and 34 for complete protocols.

### Volatility assays

To assess the evaporation of each C_5_ alcohol, 3-methyl-3-buten-1-ol, 3-methyl-2-buten-1-ol, and 3-methyl-1-butanol were spiked into 5 mL of EZ-Rich media in culture tubes (triplicate) at various concentrations and placed at 30 °C while shaking (200 rpm) for 48 hours. One set of tubes was placed at 4 °C to act as a control. Samples were collected for alcohol quantification by GC-FID at times 0, 24, and 48 hours.

### Targeted proteomics analysis

At 24 hours, 1.5 mL of production culture was collected and pelleted by centrifugation at 8000 × g (4 °C). After the supernatant was decanted, cell pellets were frozen in liquid nitrogen and stored at −80 °C. Sample preparation and protein extraction was performed as described previously^14^,^22^.

## Additional Information

**How to cite this article**: George, K. W. *et al.* Metabolic engineering for the high-yield production of isoprenoid-based C_5_ alcohols in *E. coli*. *Sci. Rep.* **5**, 11128; 10.1038/srep11128 (2015).

## Supplementary Material

Supplementary Information

## Figures and Tables

**Table 1:** **Plasmids and strains used in this study**.

**Plasmids**	**Description**	**Reference**
pJBEI-6829	pBbA5c-MevTsa-MK-PMK	14
pJBEI-6833	pTrc99A-nudB_RBSo_-PMD	14
JPUB-004498	pTrc99A-nudB_RBS1_-PMD	This study
JPUB-004499	pTrc99A-nudB_RBS2_-PMD	This study
JPUB-004500	pTrc99A-nudB_RBS3_-PMD	This study
JPUB-004501	pTrc99A-nudB_RBS4_-PMD	This study
JPUB-004502	pTrc99A-nudB_RBS5_-PMD	This study
JPUB-004503	pTrc99A-nudB_RBS6_-PMD	This study
JPUB-004504	pTrc99A-nudB_RBS7_-PMD	This study
JPUB-004505	pTrc99A-nudB_RBS8_-PMD	This study
JPUB-004506	pTrc99A-nudB_RBS9_-PMD	This study
JPUB-004507	pTrc99A-nudB_RBS10_-PMD	This study
pJBEI-6830	pBbA5c-MevTsa-PMK-MK	14
pJBEI-6832	pBbA5c-MevTsa-T1002-ptrc-PMK-MK	14
pJBEI-6823	pBbA5c-MevTco-PMK-MK	14
JPUB-004508	pBbA5c-MevTco-PMK-MKsa	This study
JPUB-004509	pBbA5c-MevTco-PMK-MKmm	This study
JPUB-004510	pBbA5c-MevTsa-PMK-MKmm	This study
JPUB-004511	pTrc99A-idi1~nudB-nemA-PMD	This study
JPUB-004512	pTrc99A-idi1_rbs10_~nudB-nemA-PMD	This study
pJBEI-6835	pBbB8k-NudB	14
**Strains**	**Description**	**Reference**
**KG1**	**pJBEI-6829 + pJBEI-6833; base strain**	14
KG1_R1_	pJBEI-6829 **+** JPUB-004498; nudB_RBS1_	This study
KG1_R2_	pJBEI-6829 + JPUB-004499; nudB_RBS2_	This study
KG1_R3_	pJBEI-6829 + JPUB-004500; nudB_RBS3_	This study
KG1_R4_	pJBEI-6829 + JPUB-004501; nudB_RBS4_	This study
KG1_R5_	pJBEI-6829 + JPUB-004502; nudB_RBS5_	This study
KG1_R6_	pJBEI-6829 + JPUB-004503; nudB_RBS6_	This study
KG1_R7_	pJBEI-6829 + JPUB-004504; nudB_RBS7_	This study
KG1_R8_	pJBEI-6829 + JPUB-004505; nudB_RBS8_	This study
KG1_R9_	pJBEI-6829 + JPUB-004506; nudB_RBS9_	This study
KG1_R10_	pJBEI-6829 + JPUB-004507; nudB_RBS10_	This study
**KG2**	**pJBEI-6832 + pJBEI-6833; increased MK expression**	This study
KG2_R3_	pJBEI-6832 + JPUB-004500; nudB_RBS3_	This study
KG2_R10_	pJBEI-6832 + JPUB-004507; nudB_RBS10_	This study
**KG3**	**pJBEI-6830 + pJBEI-6833; MK control**	This study
KG3_R3_	pJBEI-6830 + JPUB-004500; nudB_RBS3_	This study
KG3_R10_	pJBEI-6830 + JPUB-004507; nudB_RBS10_	This study
**KG4**	**pJBEI-6823 + pJBEI-6833; yeast MK**	This study
KG4_R3_	pJBEI-6823 + JPUB-004500; nudB_RBS3_	This study
KG4_R10_	pJBEI-6823 + JPUB-004507; nudB_RBS10_	This study
**KG5**	**JPUB-004509 + pJBEI-6833;** ***M. mazei*** **MK**	This study
KG5_R3_	JPUB-004509 + JPUB-004500; nudB_RBS3_	This study
KG5_R10_	JPUB-004509 + JPUB-004507; nudB_RBS10_	This study
**KG6**	**JPUB-004508 + pJBEI-6833-;** ***S. aureus*****MK**	This study
KG6_R3_	JPUB-004508 + JPUB-004500; nudB_RBS3_	This study
KG6_R10_	JPUB-004508 + JPUB-004507; nudB_RBS10_	This study
**KG7**	**JPUB-004510 + pJBEI-6833,** ***M. mazei*****MK**	This study
KG7_R3_	JPUB-004510 + JPUB-004500; nudB_RBS3_	This study
KG7_R10_	JPUB-004510 + JPUB-004507; nudB_RBS10_	This study
**KG8**	**pJBEI-6829 + JPUB-004511; mixed alcohol production**	This study
KG8_R10_	pJBEI-6829 + JPUB-004512; Idi1_RBS10_	This study
**KG9**	pJBEI-6829 + JPUB-004511 + pJBEI-6835	This study
