# Supplementary material for: Metabolic engineering for the high-yield production of isoprenoid-based C5 alcohols in E. coli
Source: Sci Rep. 2015 Jun 8;5:11128. doi: 10.1038/srep11128 (PMC4459108; doi:10.1038/srep11128)

Supplemental Material

Metabolic engineering for the high-yield production of isoprenoid-based C_5 alcohols in *E. coli*

Kevin W. George[1,2], Mitchell G. Thompson[1,3], Aram Kang[1,2], Edward Baidoo[1,2], George Wang[1,2], Leanne Jade G. Chan[1,2], Paul D. Adams[1,2], Christopher J. Petzold[1,2], Jay D. Keasling[1,2,4,5] Taek Soon Lee[1,2,*]

Supplemental Table 1. RBS sequences used in NudB engineering.

**Name**	**Sequence**	**Predicted translation initiation rate**
RBSo (from KG1)	TTTCACACAGGAAACAGACC	N/A*
RBS1	TTAAAGACGCAGATACACTT	5.42
RBS2	GTTAAGAAGCAGATACCCAT	375.41
RBS3	GTAAAGAAGCAGATACAGTT	410.76
RBS4	GGCGTAAACTACATACAGGAGGGAC	1027.83
RBS5	TTAAGACGGAGATACAGTT	1657.65
RBS6	GTACAGAAGGAGATACACTT	5341.55
RBS7	TTAAGGAAGGAGATAGACTT	8008.93
RBS8	TGACAGAAGGAGATAGAGTT	23586.28
RBS9	TTTAAGAAGGAGATATAGTT	25807.73
RBS10	TTTAAGAAGGAGATATACAT	26784.21

*RBSo was the input sequence used to generate RBS1 through RBS10

Supplemental Figure S1. Strain KG1 pathway proteomics. Samples were collected and analyzed for MVA pathway proteins in strain KG1 over a 48 hour time-course to assess stability and identify potential bottlenecks. A targeted SRM method was used (see methods). Time 0 represents the point of induction (OD=0.4). Error bars represent standard deviation (n=3).

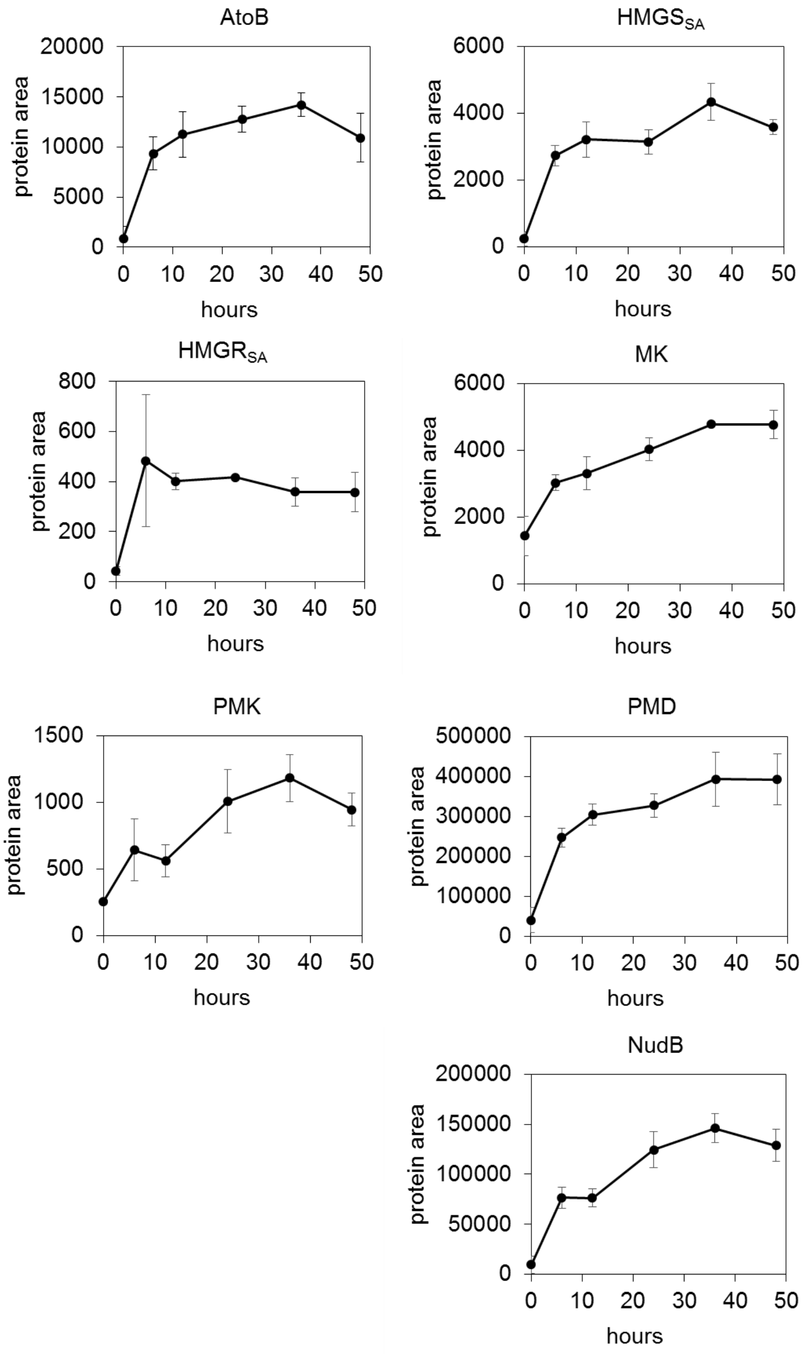

Supplemental Figure S2. Third plasmid NudB expression does not further improve titer. The introduction of a supplemental plasmid ("pNudB" or pBbB8k-NudB, see Table 1) did not improve 3-methyl-3-buten-1-ol titer in strains KG1 or KG2. This suggested that IPP accumulation was no longer a bottleneck in these strains. An empty vector was used as a control (pEmpty). Error bars show standard deviation (n=3).

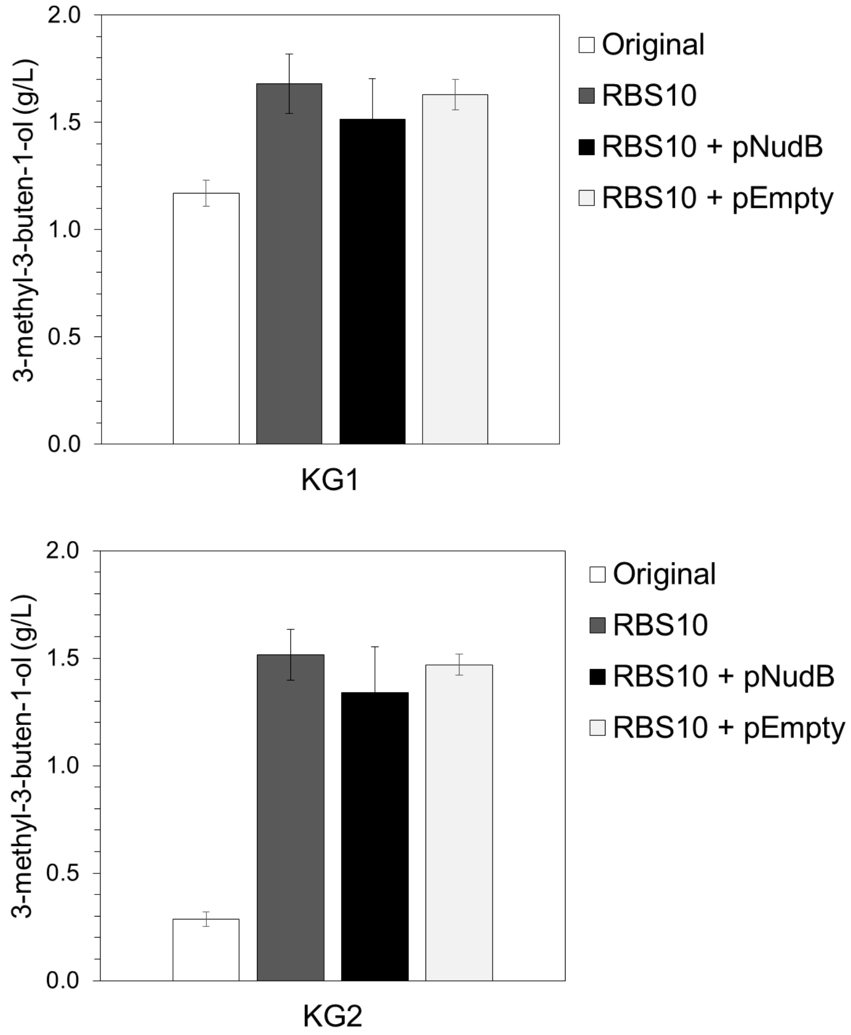

Supplemental Figure S3. Growth inhibition caused by expression of $MK_{MM.}$ Growth inhibition was clearly observed in strain KG5, containing MK from *M. mazei*. Improved NudB expression ($KG5_{R10}$) relieved this inhibition, suggesting that IPP accumulation was the primary cause of this growth defect. Note that no inhibition was observed in strains carrying MK enzymes from *S. cerevisiae* and *S. aureus* (KG4 and KG6, respectively). Error bars show standard deviation (n=3).

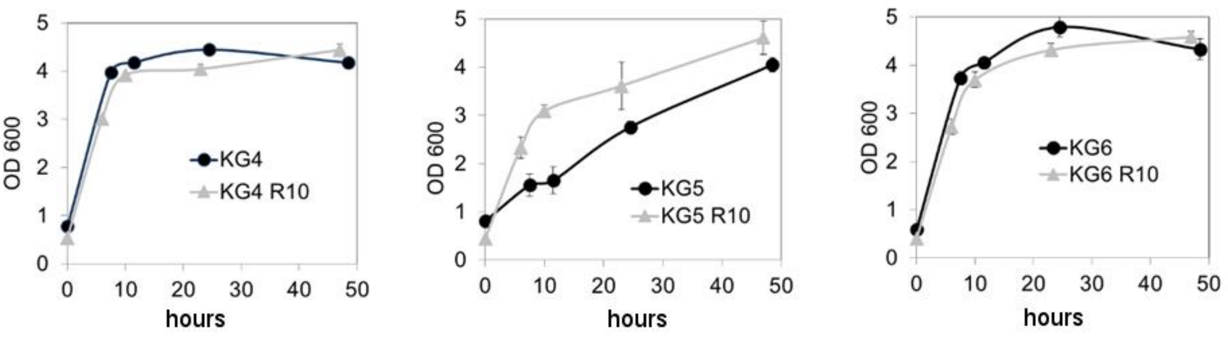

Supplemental Figure S4. Decreased recoverable 3-methyl-3-buten-1-ol in late time points. A minor decrease in recoverable 3-methyl-3-buten-1-ol was observed in strain KG1 at time points past 48 hours, suggestive of volatility or perhaps transformation. Error bars represent standard deviation (n=3).

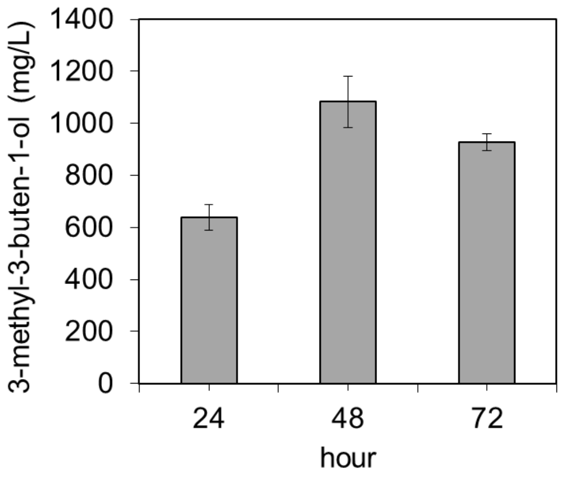

Supplemental Figure S5. Volatility of 3-methyl-3-buten-1-ol, 3-methyl-2-buten-1-ol, and 3-methyl-1-butanol. Various concentrations of each C5 alcohol were spiked into culture tubes containing 5 mL of medium and incubated at 30°C in an orbital shaker (200 rpm). A decrease in recoverable alcohol was observed in each case, indicating product loss to volatility. Control tubes that were incubated at 4°C showed no decreases in recoverable alcohol. Error bars show standard deviation (n=3).

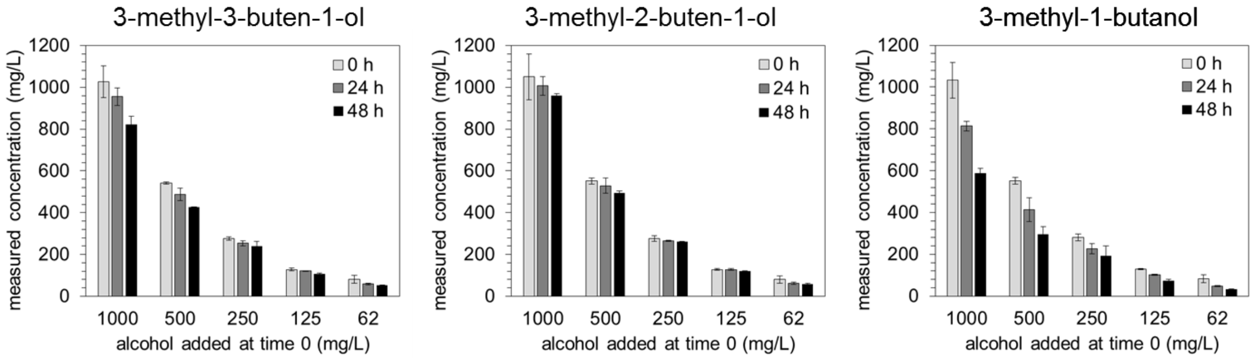

Supplemental Figure S6. Oleyl alcohol partitioning. Each alcohol was added at a concentration of 1000 mg/L to 5 mL of culture medium containing various concentrations of oleyl alcohol overlay. These solutions were shaken (200 rpm) at 30°C for 1 hour. After incubation, samples of the aqueous phase were taken and alcohols were quantified by GC-FID. 3-methyl-1-butanol partitioned most readily into the overlay. For each alcohol, increased overlay content resulted in increased partitioning into the oleyl alcohol phase. Error bars show standard deviation (n=3). A 20% overlay was used in production assays with strains $KG1_{R10}$ and KG9.

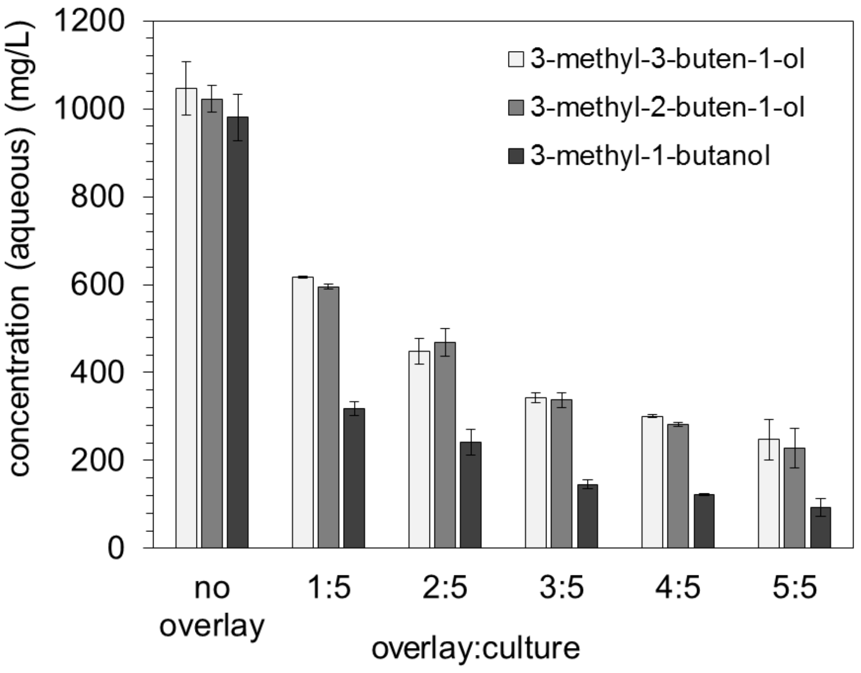

Supplement: Supplementary Information [file srep11128-s1.pdf]
